# Trans-fatty acid blood levels of industrial but not natural origin are associated with cardiovascular risk factors in patients with HFpEF: a secondary analysis of the Aldo-DHF trial

**DOI:** 10.1007/s00392-022-02143-7

**Published:** 2023-01-14

**Authors:** Katharina Lechner, Matthias Bock, Clemens von Schacky, Johannes Scherr, Elke Lorenz, Benjamin Lechner, Bernhard Haller, Alexander Krannich, Martin Halle, Rolf Wachter, André Duvinage, Frank Edelmann

**Affiliations:** 1 Klinik für Herz- und Kreislauferkrankungen, Deutsches Herzzentrum München, Technische Universität München, Munich, Germany; 2 DZHK (German Centre for Cardiovascular Research), Partner site Munich, Munich Heart Alliance, Munich, Germany; 3 Department of Prevention, Rehabilitation and Sports Medicine, School of Medicine, Technical University of Munich, Munich, Germany; 4 Omegametrix, Munich, Germany; 5 University Center for Prevention and Sports Medicine, Balgrist University Hospital, University of Zurich, Zurich, Switzerland; 6 Department of Internal Medicine IV, Ludwig-Maximilians University, Munich, Germany; 7 Institute of AI and Informatics in Medicine, Klinikum rechts der Isar, Technische Universität München, Munich, Germany; 8 Charité, Universitätsmedizin Berlin, Berlin, Germany; 9 Clinic and Policlinic for Cardiology, University Hospital Leipzig, Leipzig, Germany; 10 Department of Cardiology and Pneumology, University Medical Center Göttingen, Georg-August University, Göttingen, Germany; 11 DZHK (German Centre for Cardiovascular Research), Partner site Göttingen, Göttingen, Germany; 12 Department of Cardiology, Campus Virchow Klinikum (CVK), Charité, Universitätsmedizin Berlin, Augustenburger Platz 1, 13353 Berlin, Germany; 13 DZHK (German Centre for Cardiovascular Research), Partner site Berlin, Berlin, Germany

**Keywords:** Trans-fatty acids, Heart failure, HFpEF, Diastolic dysfunction, Atherogenic dyslipidemia, Aerobic capacity, Cardiac function

## Abstract

**Background:**

Industrially processed trans-fatty acids (IP-TFA) have been linked to altered lipoprotein metabolism, inflammation and increased NT-proBNP. In patients with heart failure with preserved ejection fraction (HFpEF), associations of TFA blood levels with patient characteristics are unknown.

**Methods:**

This is a secondary analysis of the Aldo-DHF-RCT. From 422 patients, individual blood TFA were analyzed at baseline in *n* = 404 using the HS-Omega-3-Index^®^ methodology. Patient characteristics were: 67 ± 8 years, 53% female, NYHA II/III (87/13%), ejection fraction ≥ 50%, *E*/*e*′ 7.1 ± 1.5; NT-proBNP 158 ng/L (IQR 82–298). A principal component analysis was conducted but not used for further analysis as cumulative variance for the first two PCs was low. Spearman’s correlation coefficients as well as linear regression analyses, using sex and age as covariates, were used to describe associations of whole blood TFA with metabolic phenotype, functional capacity, echocardiographic markers for LVDF and neurohumoral activation at baseline and after 12 months.

**Results:**

Blood levels of the naturally occurring TFA C16:1n-7t were inversely associated with dyslipidemia, body mass index/truncal adiposity, surrogate markers for non-alcoholic fatty liver disease and inflammation at baseline/12 months. Conversely, IP-TFA C18:1n9t, C18:2n6tt and C18:2n6tc were positively associated with dyslipidemia and isomer C18:2n6ct with dysglycemia. C18:2n6tt and C18:2n6ct were inversely associated with submaximal aerobic capacity at baseline/12 months. No significant association was found between TFA and cardiac function.

**Conclusions:**

In HFpEF patients, higher blood levels of IP-TFA, but not naturally occurring TFA, were associated with dyslipidemia, dysglycemia and lower functional capacity. Blood TFAs, in particular C16:1n-7t, warrant further investigation as prognostic markers in HFpEF.

**Graphical abstract:**

Higher blood levels of industrially processed TFA, but not of the naturally occurring TFA C16:1n-7t, are associated with a higher risk cardiometabolic phenotype and prognostic of lower aerobic capacity in patients with HFpEF.

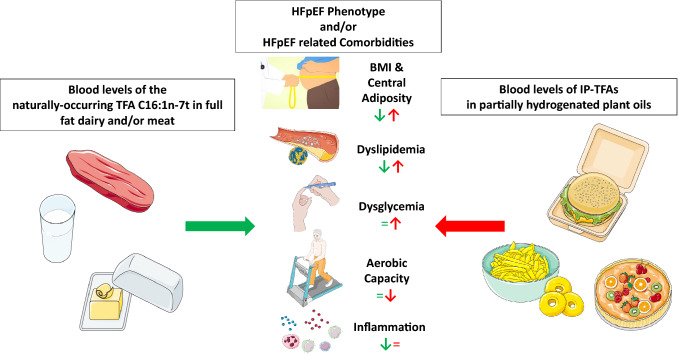

**Supplementary Information:**

The online version contains supplementary material available at 10.1007/s00392-022-02143-7.

## Introduction

Heart failure with preserved ejection fraction (HFpEF) is a heterogeneous syndrome that is closely linked to metabolic conditions (i.e., ectopic adiposity and associated metabolic traits) and comorbidities such as coronary heart disease (CHD) [1, 2]. Collinearly with the increasing prevalence of adiposity, type 2 diabetes (T2D) and hypertension, the prevalence of HFpEF has risen during the last decades and is expected to further increase due to the demographic change and/or increase in individuals living with metabolic conditions [3, 4]. The coexistence of T2D and HFpEF occurs in 30–40% of patients and is associated with a higher risk of heart failure (HF) hospitalization, all-cause as well as cardiovascular (CV) mortality [4]. This burdens health systems worldwide since HFpEF accounts for more than 50% of heart failure (HF) hospital admissions [3]. Contrary to management of heart failure with reduced ejection fraction (HFrEF), where innovations were achieved by medical and device therapy [5], medical treatment options with prognostic benefit for patients living with HFpEF are limited to sodium–glucose co-transporter-2 inhibitors (SLGT2i) [6]. Effective treatment of HFpEF thus remains an unmet clinical need that largely depends on optimization of concomitant risk factors and comorbidities by means of lifestyle interventions and/or pharmacotherapy [1, 7].

Trans fats are a heterogeneous group of unsaturated fatty acids that are chemically characterized by the presence of at least one double bond in the trans configuration [8]. Industrially processed trans-fatty acids (IP-TFA) are formed during the industrial process of partial hydrogenation of vegetable oils, i.e., the conversion of liquid vegetable oils into semisolid fats for use in margarines, commercial cooking and manufacturing processes to increase shelf life and palatability of food [8]. Naturally occurring TFA in meats and dairy products are produced by bacteria in the ruminant stomach [8]. While concordant evidence from controlled trials and observational studies show that consumption of IP-TFA (as measured by subjective memory-based methods such as food frequency questionnaires) adversely affects cardiovascular risk factors, and is a strong and independent risk factor for coronary heart disease (CHD) events [8–11], no association was found between dietary TFAs and incident HF in male physicians from the Physicians’ Health Study [12].

Regarding objective biomarkers of TFA status and their association with CHD, cardiometabolic risk factors and heart failure, evidence is heterogeneous. While two studies reported an increased odds of acute coronary syndromes in those with higher blood cell content of IP-TFA [13, 14], higher blood/red blood cell (RBC) trans FAs were no independent predictor of increased risk for acute coronary syndromes [15] or mortality in another study [16]. Regarding metabolic risk factors, Lee et al. [17] report significant positive associations between RBC TFAs and waist circumference/BMI in a case–control study in Koreans. Contrarily, a pooled analysis of 12 prospective cohort studies of the Fatty Acids and Outcomes Research Consortium (FORCE) showed no association of T2D risk with circulating individual trans-18:2 TFA biomarkers and an inverse association of T2D risk with trans-16:1n-9, total trans-18:1 and total trans-18:2 [18]. Regarding HF, Tokede et al. [12] described a lower risk of HF with higher concentrations of the biomarker plasma trans-fatty acid C18:2 in male physicians from the Physicians’ Health Study. In patients with established HF, RBC TFAs were strongly associated with high inflammatory burden such as higher levels of interleukin (IL) 1β, IL-1 receptor antagonist, IL-6, IL-10, tumor necrosis factor (TNF), TNF receptor 1 and 2, monocyte chemoattractant protein 1 and brain natriuretic peptide (BNP) [19].

In patients with HFpEF specifically, associations between individual blood trans-fatty acid levels with patient characteristics are unknown. We thus investigated associations of blood TFA with metabolic phenotype, functional capacity, echocardiographic markers indicative of left ventricular diastolic function (LVDF) and neurohumoral activation in a large cohort comprised of 404 HFpEF patients from the Aldosterone in Diastolic Heart Failure (Aldo-DHF) trial. As TFA are not endogenously synthesized, blood levels of TFA are a biomarker for the balance of dietary TFA intake, distribution volume and catabolism independent of subjective memory-based assessment methods such as food frequency questionnaires [20]. They—like other blood fatty acid levels such as EPA and DHA levels [21]—reliably reflect cardiac and other tissue TFA levels over approximately the preceding 3 months [22, 23].

We hypothesized that blood IP-TFA would directly correlate with cardiovascular risk factors, aerobic capacity and left ventricular diastolic function (LVDF), and neurohumoral activation in patients with HFpEF.

## Methods

### Study design

This study is a post hoc analysis of the Aldo-DHF trial (ISRCTN 94726526). Baseline blood levels of TFA were analyzed. 18 whole blood aliquots were not available due to loss during storage/transfer or missing blood sampling at baseline of a total of 422 patients enrolled in the Aldo-DHF trial as described in our previous analysis of n-3 fatty acids in this cohort [21].

### The Aldo-DHF trial

The Aldo-DHF trial is a multicenter, prospective, randomized, double-blind, placebo-controlled trial evaluating the effect of a 12-month aldosterone receptor blockade on diastolic function (*E*/*e*′) on echocardiography and maximal exercise capacity (*V*O_2peak_) in HFpEF patients. Men and women aged 50 years or older were eligible to participate in the study if they had current HF symptoms consistent with New York Heart Association (NYHA) class II or III, left ventricular ejection fraction (LVEF) of 50% or greater, echocardiographic evidence of diastolic dysfunction (grade I) or atrial fibrillation at presentation and maximal exercise capacity (VO2peak) of 25 mL/kg/min or less [24]. 422 ambulatory patients (mean age 67 [SD, 8] years; 52% female) with evidence of diastolic dysfunction were included. Data acquisition was conducted between March 2007 and April 2012 at 10 sites in Germany and Austria [24].

### Laboratory measurements

#### Laboratory methods: Aldo-DHF trial

Venous blood samples were drawn under standardized conditions after 20 min of rest in supine position. Samples were immediately cooled, centrifuged and processed for storage at − 80 °C (− 112 °F). N-terminal pro-braintype natriuretic peptide (NT-proBNP) was analyzed with the Elecsys NT-proBNP immunoassay (Roche Diagnostics) [24].

#### Laboratory methods: HS-Omega-3 Index^®^ methodology

Blood samples from the Aldo-DHF trial were immediately stored at − 80 °C. This results in stable levels of blood fatty acids [25]. For the analysis of fatty acid composition, 2.0 mL aliquots of frozen (− 80 °C) EDTA blood was sent to Omegametrix (Martinsried, Germany). At Omegametrix, a reference laboratory for fatty acid analyses, whole blood fatty acid composition was analyzed according to the HS-Omega-3 Index^®^ methodology [26]. Fatty acid methyl esters were generated by acid transesterification and were analyzed by gas chromatography using a GC2010 Gas Chromatograph (Shimadzu, Duisburg, Germany) equipped with a SP2560, 100-m column (Supelco, Bellefonte, Pennsylvania, USA) using hydrogen as carrier gas. Fatty acids were identified by comparison with a standard mixture of fatty acids characteristic of erythrocytes. Individual fatty acid results are given as relative amounts of C16:1n-7t, C18:1n9t, C18:2n6tt, C18:2n6ct and C18:2n6tc expressed as a percentage of a total of 26 identified FAs in whole blood. Analyses were quality-controlled according to DIN ISO 15189.

#### Echocardiography and other variables

In the Aldo-DHF Trial, clinical data were obtained and diagnostic procedures were done according to predefined standard operating procedures based on international guidelines [24]. Diastolic function on echocardiography was assessed in accordance with American Society of Echocardiography guidelines [27].

### Ethics

The Aldo-DHF Trial complies with the Declaration of Helsinki and principles of good clinical practice. All responsible ethics committees approved the study protocol. All participants gave written informed consent.

### Statistical analysis

Continuous variables are reported as mean ± standard deviation (SD) or median and interquartile range (IQR), according their scale and distribution. Discrete variables are presented as absolute and relative frequencies. Spearman’s correlation coefficient as well as multiple linear regression analyses, using sex and age as additional covariates, were used to describe the association of whole blood TFA with cardiometabolic risk markers, echocardiographic markers of LVDF and neurohumoral activation at baseline and at 12-month follow-up (12mFU). To account for the randomization group, all analysis were repeated as sensitivity analysis with group as covariate. Furthermore, we adjusted correlations with BMI, markers for truncal adiposity (waist circumference and waist-to-height ratio), HbA1c and systolic/diastolic blood pressure, by partial correlation. A principal component analysis (PCA) was conducted to consolidate variables due to the limited sample size. A significance level of *α* = 5% was used for all tests. As all tests were hypothesis generating without confirmatory interpretation, no correction for multiple testing was applied. All statistical analyses were performed using R and RStudio, version R-4.2.125 (R Foundation for Statistical Computing, Vienna, Austria).

## Results

### Study population

**Fig. 1:**
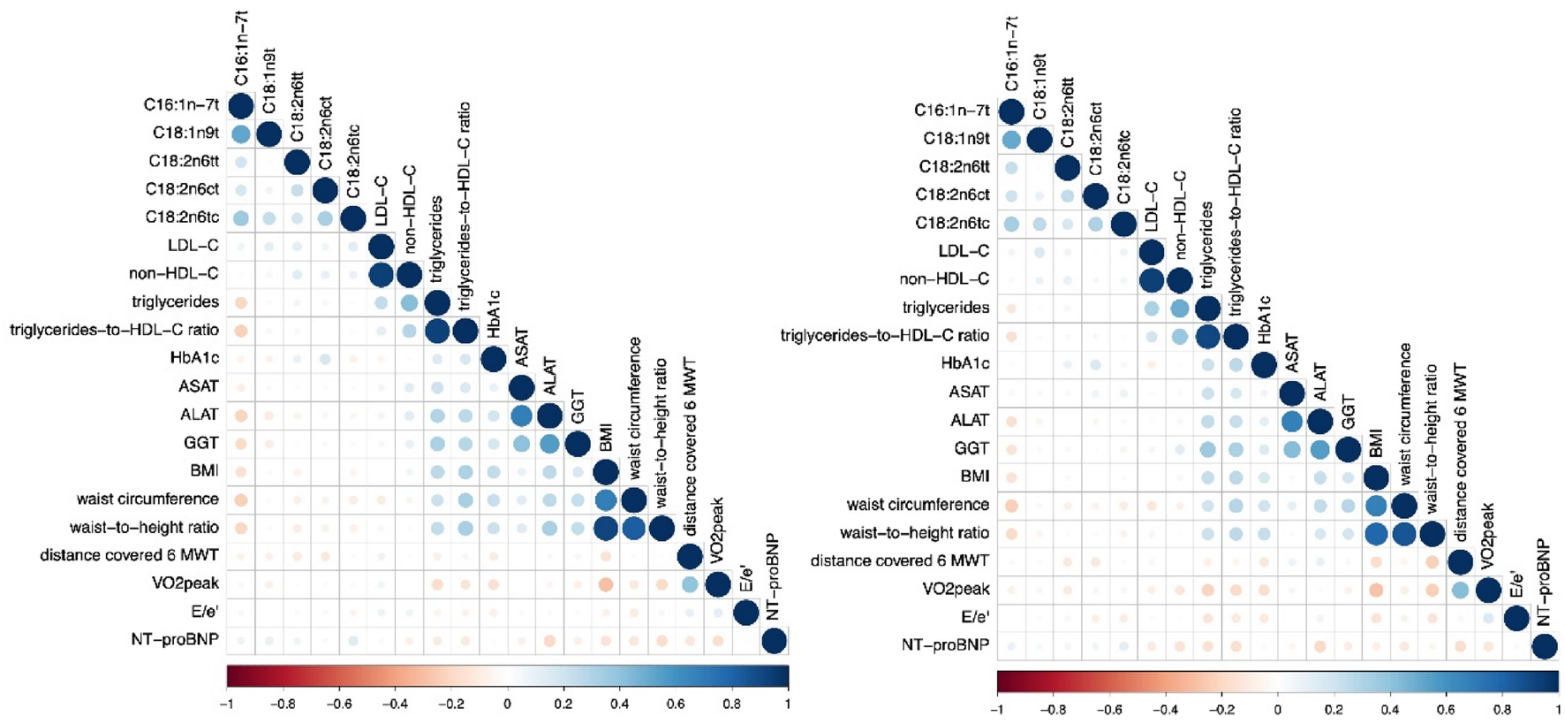
Correlation plot showing associations between individual TFA and patient characteristics at baseline (left) and 12-month follow-up (right). Blue color indicates a positive association, and orange color indicates an inverse association. Higher color intensity and larger circles indicate a stronger association, lower color intensity and smaller circles indicate a weaker association. *NT-proBNP* N-terminal pro-braintype natriuretic peptide, *GGT* γ-glutamyltransferase, *ASAT* aspartate aminotransaminase, *ALAT* alanine aminotransaminase, *E*/*e*′ diastolic function, *VO*_*2peak*_ maximum exercise capacity

Baseline characteristics are shown in Table 1. Tables 2 and 3 and Fig. 1 depict the associations of individual TFA [i.e., the naturally occurring C16:1n-7t (trans-palmitoleic acid) and the industrially processed TFA C18:1n9t, C18:2n6tt, C18:2n6ct and C18:2n6tc] with patient characteristics at baseline and 12mFU, respectively. Statistically significant associations with clinical relevance are reported in the text, and all associations are depicted in Tables 2 and 3.

**Table 1:** Baseline characteristics

Characteristics^a^	Total (*n* = 404)
Demographics	
Age, mean (SD) (years)	67 (8)
Female	212 (53)
Laboratory measures	
HbA1c (%)	6.0 (0.8)
LDL-C (mg/dl)	117 (42)
HDL-C (mg/dl)	56 (18)
Triglycerides (mg/dl)	161 (103)
Non-HDL-C (mg/dl)	133 (47)
TG/HDL-C ratio	3.3 (2.8)
NT-proBNP, median (IQR) (ng/L)	158 (82–298)
Naturally occurring TFA (%)	
C16:1n-7t	0.14 (0.04)
IP-TFA (%)	
C18:1n9t	0.51 (0.97)
C18:2n6tt	0.01 (0.01)
C18:2n6ct	0.04 (0.02)
C18:2n6tc	0.12 (0.03)
Medical history	
Hospitalization for heart failure in past 12 months^c^	149 (37)
Hypertension	370 (92)
Diabetes mellitus	66 (16)
Atrial fibrillation	65 (16)
Physical examination, mean (SD)	
Body mass index^b^	28.9 (3.6)
Waist circumference (cm)	98.1 (11.0)
In men	103.7 (9.0)
In women	93.1 (10.3)
Waist-to-height ratio	0.49 (0.1)
Systolic blood pressure (mmHg)	135 (18)
Diastolic blood pressure (mmHg)	79 (11)
Heart rate (/min)	66 (11)
Signs and symptoms	
NYHA functional class	
II	350 (87)
III	54 (13)
Peripheral edema	160 (40)
Nocturia	325 (80)
Paroxysmal nocturnal dyspnea	66 (16)
Nocturnal cough	61 (15)
Fatigue	241 (60)
Current medications	
ACE inhibitors/angiotensin receptor antagonists	310 (77)
Betablockers	290 (72)
Diuretics	213 (53)
Calcium antagonists	97 (24)
Lipid-lowering drugs	221 (55)
Echocardiography, mean (SD)	
LV ejection fraction (%)	68 (8)
LV diameter (end diastolic) (mm)	46.5 (6.2)
LV diameter (end systolic) (mm)	25.3 (6.4)
LV mass index (g/m^2^)	114.15 (45.53)
Left atrial volume index (mL/m^2^)	43.1 (41.6)
E-wave velocity (cm/s)	73 (20)
Medial *e*′ wave velocity (cm/s)	5.9 (1.3)
*E*/*e*′	7.1 (1.5)
*E*/*A* velocity ratio	0.91 (0.33)
Isovolumic relaxation time (ms)	88 (26)
Deceleration time (ms)	243 (63)
Grade of diastolic dysfunction, no. (%)	
I	295 (73)
II	81 (20)
III	4 (1)
IV	3 (1)

*ACE* angiotensin-converting enzyme, *IQR* interquartile range, *NT-proBNP* N-terminal pro-braintype natriuretic peptide, *NYHA* New York Heart Association, *A* peak atrial transmitral ventricular filling velocity, *e*′ early diastolic tissue Doppler velocity, *E* peak early transmitral ventricular filling velocity

^a^Data are expressed as no. (%) unless otherwise specified

^b^Body mass index is defined as weight in kilograms divided by height in meters squared

^c^2 missing from analysis (*n* = 402)

**Table 2:** Associations between individual TFA and patient characteristics at baseline

	C16:1n-7t (trans-palmitoleic acid)	C18:1n9t	C18:2n6tt	C18:2n6ct	C18:2n6tc
LDL-C					
*r*^§^	0.067	**0.118**	**0.118**	0.069	**0.13**
*p*-value*	0.196	**0.023**	**0.023**	0.182	**0.012**
Non-HDL-C					
*r*^§^	− 0.015	0.043	**0.121**	0.092	0.083
*p*-value*	0.78	0.414	**0.02**	0.077	0.109
Triglycerides					
*r*^§^	**− 0.201**	0.023	0.051	0.044	0.01
*p*-value*	**< 0.001**	0.659	0.329	0.398	0.846
Triglycerides-to-HDL-C ratio					
*r*^§^	**− 0.23**	0.035	0.038	0.003	0.016
*p*-value*	**< 0.001**	0.499	0.464	0.954	0.76
HbA1c					
*r*^§^	− 0.062	− 0.064	0.086	**0.163**	− 0.071
*p*-value*	0.233	0.216	0.096	**0.002**	0.172
ASAT					
*r*^§^	− 0.086	− 0.031	− 0.019	− 0.026	0.028
*p*-value*	0.1	0.546	0.721	0.624	0.587
ALAT					
*r*^§^	**− 0.217**	**− 0.105**	− 0.061	− 0.05	− 0.048
*p*-value*	**< 0.001**	**0.043**	0.244	0.334	0.357
GGT					
*r*^§^	**− 0.184**	− 0.096	− 0.011	0.03	− 0.026
*p*-value*	**< 0.001**	0.064	0.834	0.559	0.616
BMI					
*r*^§^	**− 0.159**	− 0.024	− 0.065	− 0.004	− 0.055
*p*-value*	**0.002**	0.642	0.212	0.933	0.287
Waist circumference					
*r*^§^	**− 0.236**	− 0.027	− 0.082	− 0.066	− 0.1
*p*-value*	**< 0.001**	0.6	0.117	0.203	0.054
Waist-to-height ratio					
*r*^§^	**− 0.207**	− 0.027	− 0.097	− 0.064	− 0.08
*p*-value*	**< 0.001**	0.605	0.061	0.22	0.122
Distance covered 6 MWT					
*r*^§^	− 0.07	− 0.073	**− 0.11**	**− 0.114**	− 0.029
*p*-value*	0.18	0.159	**0.034**	**0.029**	0.575
*V*O_2peak_					
*r*^§^	− 0.037	− 0.078	− 0.012	0.025	0.023
*p*-value*	0.472	0.133	0.812	0.625	0.654
*E*/*e*′					
*r*^§^	− 0.039	− 0.007	− 0.022	− 0.032	− 0.056
*p*-value*	0.449	0.887	0.668	0.539	0.281
NT-proBNP					
*r*^§^	0.078	0.099	0.062	0.032	**0.139**
*p*-value*	0.135	0.056	0.234	0.537	**0.007**

Significant values are in bold

*NT-proBNP* N-terminal pro-braintype natriuretic peptide, *GGT* γ-glutamyltransferase, *ASAT* aspartate aminotransaminase, *ALAT* alanine aminotransaminase, *E*/*e*′ diastolic function, *VO*_*2peak*_ maximum exercise capacity

*All tests were performed two-sided. *r*^§^ (Spearman’s correlation coefficient)

**Table 3:** Associations between individual TFA and patient characteristics at 12mFU

	C16:1n-7t (trans-palmitoleic acid)	C18:1n9t	C18:2n6tt	C18:2n6ct	C18:2n6tc
LDL-C					
*r*^§^	0.06	**0.158**	0.063	0.015	0.072
*p*-value*	0.269	**0.003**	0.248	0.78	0.187
Non-HDL-C					
*r*^§^	0.034	0.082	0.098	0.041	0.054
*p*-value*	0.53	0.13	0.071	0.446	0.318
Triglycerides					
*r*^§^	− **0.121**	0.01	0.042	0.008	0.004
*p*-value*	**0.025**	0.853	0.437	0.884	0.941
Triglycerides-to-HDL-C ratio					
*r*^§^	− **0.151**	− 0.013	0.029	− 0.031	− 0.019
*p*-value*	**0.005**	0.804	0.59	0.572	0.722
HbA1c					
*r*^§^	0.01	0.004	0.087	**0.157**	− 0.039
*p*-value*	0.851	0.946	0.109	**0.004**	0.475
ASAT					
*r*^§^	− 0.04	0.038	0.039	0.069	0.079
*p*-value*	0.462	0.483	0.472	0.201	0.147
ALAT					
*r*^§^	− **0.153**	− 0.054	0.002	− 0.042	− 0.033
*p*-value*	**0.005**	0.324	0.97	0.436	0.538
GGT					
*r*^§^	− **0.143**	− 0.032	0.031	0.013	− 0.051
*p*-value*	**0.008**	0.556	0.564	0.81	0.35
BMI					
*r*^§^	− **0.145**	0.004	− 0.032	− 0.002	− 0.023
*p*-value*	**0.007**	0.942	0.553	0.969	0.666
Waist circumference					
*r*^§^	− **0.24**	− 0.039	− 0.077	− 0.064	− 0.097
*p*-value*	**< 0.001**	0.469	0.156	0.24	0.073
Waist-to-height ratio					
*r*^§^	− **0.186**	− 0.056	− 0.022	0.025	− 0.063
*p*-value*	**0.001**	0.302	0.69	0.64	0.249
Distance covered 6 MWT					
*r*^§^	− 0.031	0.024	− **0.113**	− **0.119**	0.021
*p*-value*	0.567	0.654	**0.036**	**0.028**	0.697
*V*O_2peak_					
*r*^§^	− 0.075	− 0.048	− 0.106	− 0.044	0.047
*p*-value*	0.17	0.379	0.051	0.413	0.382
*E*/*e*′					
*r*^§^	− 0.009	− 0.027	− 0.041	− 0.09	− 0.097
*p*-value*	0.873	0.614	0.447	0.098	0.073
NT-proBNP					
*r*^§^	0.082	0.046	0.058	0.066	**0.113**
*p*-value*	0.133	0.401	0.289	0.225	**0.038**

Significant values are in bold

*NT-proBNP* N-terminal pro-braintype natriuretic peptide, *GGT* γ-glutamyltransferase, *ASAT* aspartate aminotransaminase, *ALAT* alanine aminotransaminase, *E*/*e*′ diastolic function, *VO*_*2peak*_ maximum exercise capacity

*All tests were performed two-sided. *r*^§^ (Spearman’s correlation coefficient)

### Individual TFA

#### Naturally occurring TFA C16:1n-7t

**Fig. 2:**
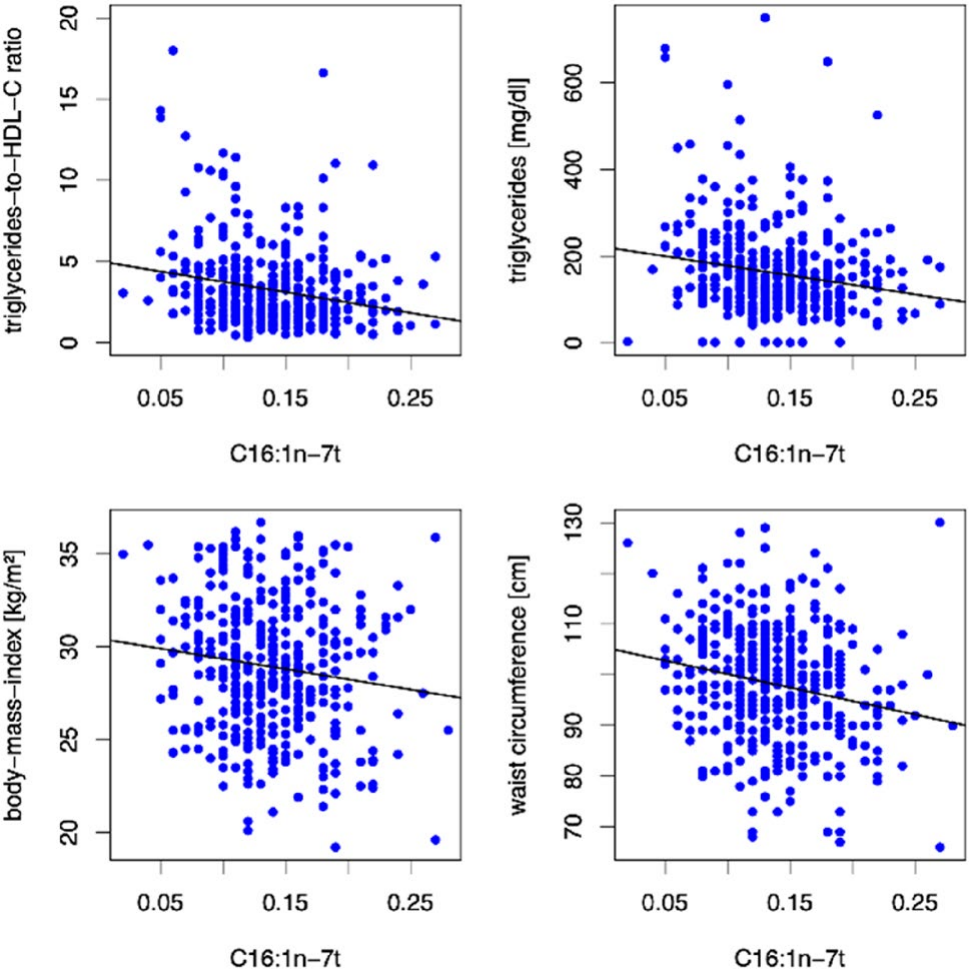
Scatter Plots showing correlations between the TFA C16:1n-7t and triglycerides-to-HDL-C ratio, triglycerides, body mass index and waist circumference at baseline. The TFA C16:1n-7t (expressed as a percentage of a total of 26 identified FAs in whole blood) was inversely associated with triglycerides-to-HDL-C ratio, triglycerides, body mass index and waist circumference at baseline in linear regression models using age and sex as covariates

As shown in Fig. 2, higher blood levels of the naturally occurring C16:1n-7t were inversely associated with biochemical markers for atherogenic dyslipidemia (*β* = − 11.38, *p* = 0.001 for triglycerides-to-HDL-C ratio and *β* = − 447.47, *p* < 0.001 for triglycerides) and adiposity [body mass index (*β* = − 9.61, *p* = 0.026) and waist circumference (*β* = − 26.96, *p* = 0.022)] at baseline. Furthermore, we observed an inverse association with white blood cell count (*β* = − 5.01, *p* = 0.034) and liver enzymes indicative for non-alcoholic fatty liver disease [alanine transaminase (*β* = − 35.36, *p* = 0.043) and γ-glutamyltransferase (*β* = − 110.19, *p* = 0.022)] at baseline. These associations persisted after 12-month follow-up (12mFU). Higher whole blood levels of C16:1n-7t at baseline were prognostic for lower triglycerides (*β* = − 337.07, *p* = 0.022), lower body mass index (*β* = − 11.44, *p* = 0.014), lower waist circumference (*β* = − 31.56, *p* = 0.013), lower waist-to-height ratio (*β* = − 0.23, *p* = 0.003), lower white blood cell count (*β* = − 4.76, *p* = 0.080) and lower γ-glutamyltransferase (*β* = − 150.27, *p* = 0.001) at 12mFU.

#### IP-TFA

Blood levels of the IP-TFA C18:1n9t (*β* = 5.4, *p* = 0.011) and C18:2n6tc (*β* = 151.8, *p* = 0.015) were positively associated with LDL-C levels, whereas higher blood IP-TFA C18:2n6tt and C18:2n6ct were associated with higher HbA1c (*β* = 14.59, *p* = 0.003 and *β* = 4.18, *p* = 0.014, respectively) at baseline. Furthermore, higher blood levels of the IP-TFA C18:2n6tt (*β* = − 1367.87, *p* = 0.006) and C18:2n6ct (*β* = − 361.42, *p* = 0.039) were associated with lower functional capacity at baseline and after 12mFU. Correlation coefficients are depicted in Table 2.

##### C18:1n9t

**Fig. 3:**
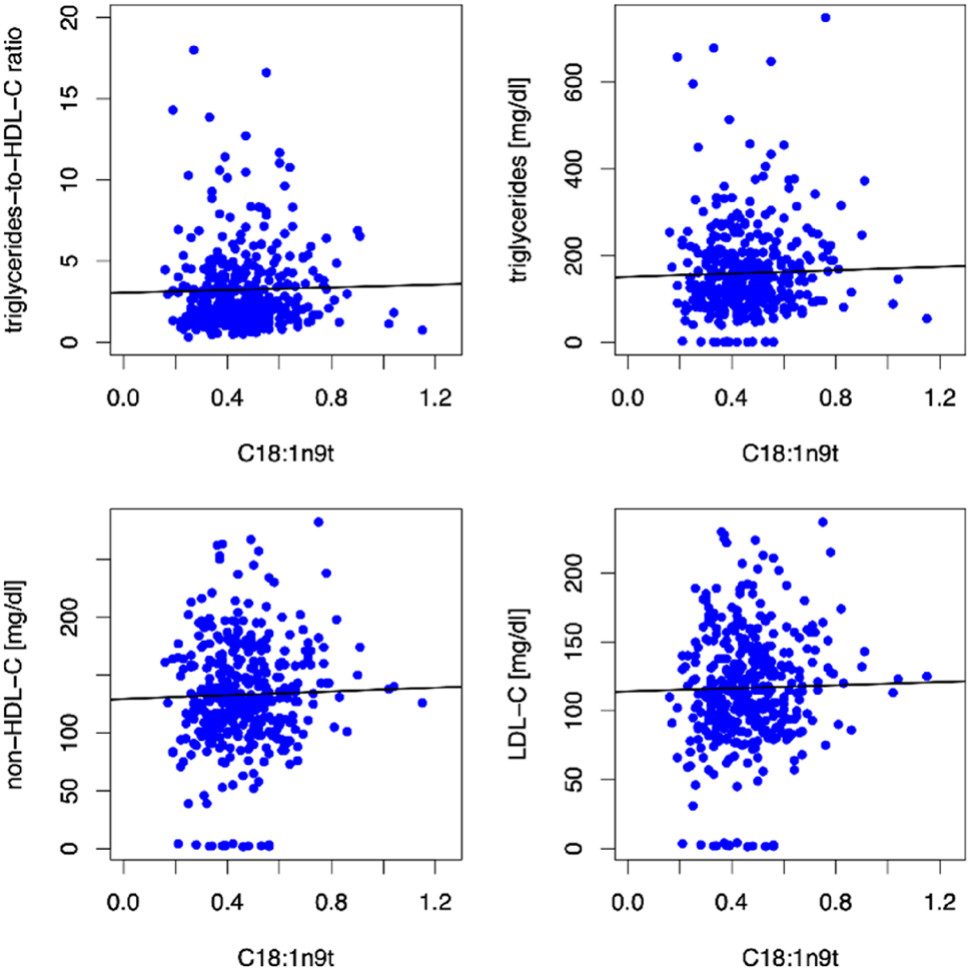
Scatter plots showing correlations between the TFA C18:1n9t and triglycerides-to-HDL-C ratio, triglycerides, non-HDL-C and LDL-C at baseline. The TFA C18:1n9t (expressed as a percentage of a total of 26 identified FAs in whole blood) was directly associated with triglycerides-to-HDL-C ratio, triglycerides, non-HDL-C and LDL-C at baseline in linear regression models using age and sex as covariates

Using linear regression models adjusted for age and sex, blood levels of industrially processed trans-fatty acids (IP-TFA) were broadly associated with high-risk traits of adiposity related HFpEF, in particular dyslipidemia. As shown in Fig. 3, higher blood levels of the IP-TFA C18:1n9t were associated with higher triglycerides-to-HDL-C ratio (*β* = 0.44, *p* = 0.002), higher triglycerides (*β* = 19.69, *p* < 0.001), higher non-HDL-C (*β* = 7.89, *p* = 0.001) and higher LDL-C (*β* = 5.4, *p* = 0.011) at baseline.

##### C18:2n6tt and C18:2n6ct

Higher blood IP-TFA C18:2n6tt and C18:2n6ct were associated with higher HbA1c (*β* = 14.59, *p* = 0.003 and *β* = 4.18, *p* = 0.014, respectively) at baseline using linear regression models adjusted for age and sex. Furthermore, higher blood levels of both C18:2n6 isomers, C18:2n6tt and C18:2n6ct, showed an association with lower submaximal aerobic capacity at baseline and after 12mFU (*β* = − 1332.6, *p* = 0.045 and *β* = − 355.39, *p* = 0.093, respectively).

##### C18:2n6tc

Similar to the IP-TFA C18:1n9t, higher blood levels of the C18:2n6 isomer C18:2n6tc were associated with dyslipidemia [non-HDL-C (*β* = 151.15, *p* = 0.029) and LDL-C (*β* = 151.8, *p* = 0.015)] at baseline.

No consistent pattern was found regarding the association between blood levels of the naturally occurring TFA and/or IP-TFA isomers with echocardiographic markers for left ventricular diastolic function. Higher blood levels of one of the three IP-TFA trans linoleic (C18:2n6) isomers (C18:2n6tc) was associated with higher levels of NT-proBNP at baseline and after 12mFU.

##### Tertile group composite C18:2n6tt, C18:2n6tc, C18:2n6ct

Additionally, as has been done previously by Kleber et al. [16] we built a composite of the three trans linoleic (C18:2n6) isomers and compared baseline characteristics of individuals with highest levels of these IP-TFA (highest tertile) compared to lower levels (lowest tertile). We observed that baseline characteristics in the highest vs the lowest tertile of the composite of the three trans linoleic (C18:2n6) isomers were different for LDL-C, non-HDL-C, waist circumference and distance covered during the 6 MWT as depicted in Table 4. LDL-C [109.68 (43.84) vs 121.00 (39.02); *p* = 0.030] and non-HDL-C [125.65 (48.66) vs 139.14 (43.11); *p* = 0.037] were higher in the group with higher levels of C18:2n6 isomers, while distance covered during the 6 MWT [543.72 (78.72) vs 515.23 (94.02); *p* = 0.026] was higher with lower levels of the C18:2n6 isomers. This further substantiates our findings as described above that higher blood levels of the C18:2n6 isomer C18:2n6tc were associated with dyslipidemia and blood levels of isomers C18:2n6tt and C18:2n6ct showed an inverse association with submaximal aerobic capacity at baseline.

**Table 4:** Descriptive table of patient characteristics at baseline in tertile groups of the three trans linoleic isomers C18:2n6tt, C18:2n6tc, C18:2n6ct as composite

	Tertile group composite (C18:2n6tt, C18:2n6tc, C18:2n6ct)			*p*
	Low	Medium	High	
*n*	150	139	115	
LDL-C [mean (SD)]	109.68 (43.84)	121.41 (41.34)	121.00 (39.02)	**0.030**
Non-HDL-C [mean (SD)]	125.65 (48.66)	137.25 (48.09)	139.14 (43.11)	**0.037**
Triglycerides [mean (SD)]	157.88 (105.70)	164.92 (102.74)	161.18 (101.93)	0.848
Triglycerides-to-HDL-C ratio [mean (SD)]	3.21 (2.76)	3.35 (2.65)	3.22 (3.16)	0.894
HbA1c [mean (SD)]	5.95 (0.73)	5.95 (0.74)	6.06 (0.80)	0.396
ASAT [mean (SD)]	25.25 (9.71)	26.65 (9.53)	26.34 (9.53)	0.429
ALAT [mean (SD)]	26.16 (14.63)	27.43 (15.52)	25.84 (14.30)	0.655
GGT [mean (SD)]	36.76 (32.45)	39.41 (51.48)	36.45 (31.66)	0.800
BMI [mean (SD)]	28.85 (3.49)	29.07 (3.55)	28.88 (3.78)	0.856
Waist circumference [mean (SD)]	98.52 (10.22)	99.42 (11.24)	96.03 (11.43)	**0.042**
Waist-to-height ratio [mean (SD)]	0.49 (0.07)	0.49 (0.06)	0.48 (0.07)	0.163
Distance covered 6 MWT [mean (SD)]	543.72 (78.72)	532.41 (82.58)	515.23 (94.02)	**0.026**
*V*O_2peak_ [mean (SD)]	16.24 (3.48)	16.47 (3.31)	16.46 (3.46)	0.818
*E*/*e*′ [mean (SD)]	7.13 (1.41)	7.12 (1.66)	6.97 (1.57)	0.674
NT-proBNP [mean (SD)]	2.15 (0.45)	2.20 (0.44)	2.27 (0.44)	0.079

Significant values are in bold

*NT-proBNP* N-terminal pro-braintype natriuretic peptide, *GGT* γ-glutamyltransferase, *ASAT* aspartate aminotransaminase, *ALAT* alanine aminotransaminase, *E*/*e*′ diastolic function, *VO*_*2peak*_ maximum exercise capacity

### Sensitivity analyses

Sensitivity analyses with medication group as additional independent variable within the linear regressions showed no significance regarding blood lipid profile, markers for (central) adiposity, aerobic capacity or liver enzymes. Therefore, group allocation (spironolactone^+/−^) had no effect on the association between these variables and TFA blood levels at 12mFU. As expected regarding the pharmacological effects of spironolactone, sensitivity analyses with medication group as covariate showed significant effects of group allocation for the association between TFA and the 12mFU outcomes: systolic/diastolic blood pressure (*p* < 0.001 for all), heart rate (only C16:1n-7t and C18:1n9t), *E*/*e*′ and HbA1c. Furthermore, all correlations were adjusted for BMI, markers for truncal adiposity (waist circumference and waist-to-height ratio), HbA1c as well as systolic/diastolic blood pressure at baseline and 12mFU (Supplemental Tables 1 and 2). These adjustments did not alter our results at 12mFU. At baseline, after adjustment for the above variables, correlations between submaximal aerobic capacity and the two trans linoleic (C18:2n6) isomers (C18:2n6tt and C18:2n6ct); alanine transaminase and C18:1n9t (*r* = − 0.09, *p* = 0.095); and γ-glutamyltransferase and C16:1n-7t (*r* = − 0.1, *p* = 0.06) were not significant anymore. However, it is of importance that the overall pattern described, in particular the association of blood trans-fatty acid status and lipid profile, was not altered by adjusting for BMI, truncal adiposity, HbA1c and blood pressure.

### Principal component analysis

**Fig. 4:**
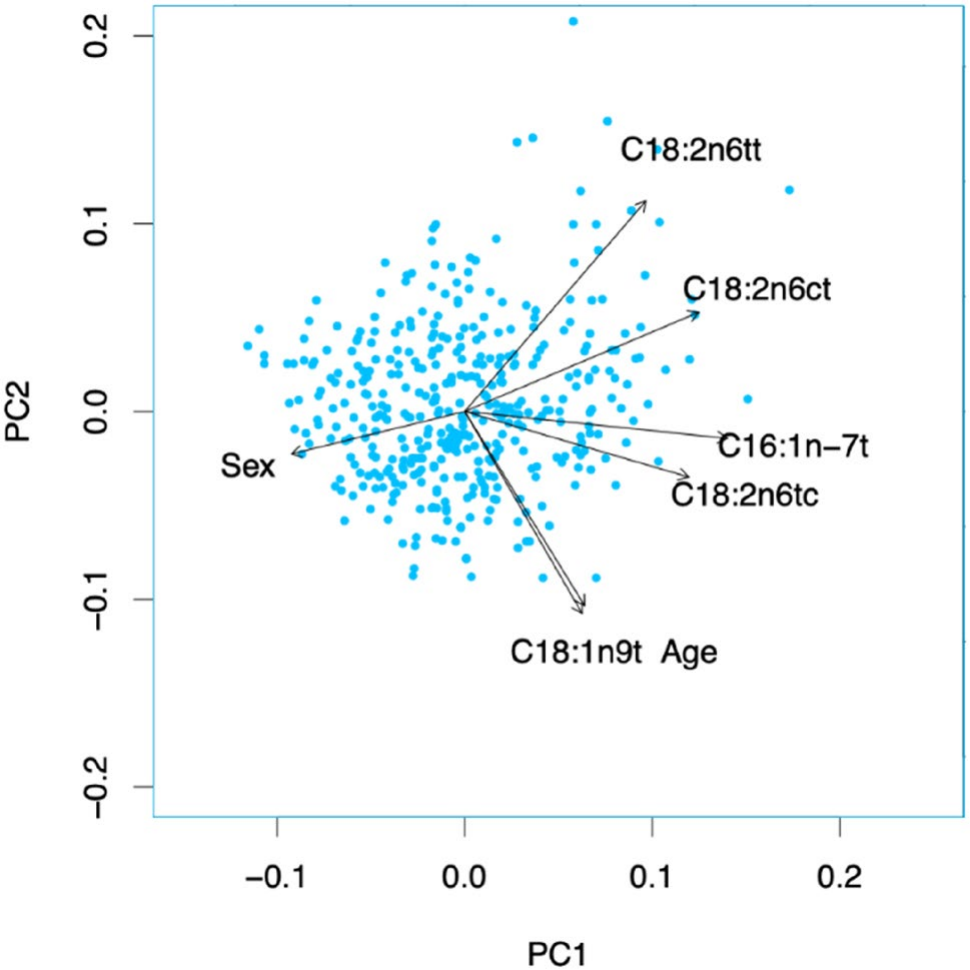
Principal component analysis (PCA). Principal component analysis from the investigated trans-fatty acids, biomarkers, age and sex. The first and second PCs are shown as bi-plot. Principal component 1 (PC1) and principal component 2 (PC2) show a relatively low cumulative variance for the first two PCs with 0.42

We reduced dimensions by using a principal component analysis (PCA) to account for the number variables in relation to the present sample size. The created PCAs show a relatively low cumulative variance for the first two PCs. Therefore, these PCs were not used in further analyses. The first and second PCs are shown as bi-plot in Fig. 4 to visualize the similarities and differences between the used predictors.

## Discussion

### Main findings

We investigated the association of TFA blood levels with cardiometabolic phenotype, aerobic capacity and cardiac function in patients with HFpEF. Using Spearman’s correlation coefficient and multiple linear regression analyses, we broadly identified two groups of TFA that had opposing associations with risk factors in HFpEF patients as depicted in the Graphical Abstract: the naturally occurring TFA C16:1n-7t was associated with a lower risk cardiometabolic phenotype while the IP-TFA trans-fatty acid C18:1n9t and the three IP-trans-18:2 isomers C18:2n6tt, C18:2n6ct and C18:2n6tc were broadly associated with cardiometabolic risk factors/HFpEF comorbidities and lower functional capacity. Further substantiating these findings, the highest versus the lowest tertile group of the composite of the three trans linoleic (C18:2n6) isomer blood levels had higher LDL-C, higher non-HDL-C and lower submaximal aerobic capacity at baseline. These associations, in particular the association of blood trans-fatty acid status and lipid profile, were not altered by adjusting for BMI, markers for truncal adiposity (waist circumference and waist-to-height ratio), HbA1c and blood pressure.

#### Naturally occurring TFA C16:1n-7t

The naturally occurring TFA C16:1n-7t occurs in a wide variety of animal products, major sources being full fat dairy and meat [28]. In the Aldo-DHF cohort, mean whole blood percentages of C16:1n-7t, expressed as a percentage of a total of 26 identified FAs, were 0.14%. There are no comparable data available on whole blood TFA concentrations in HFpEF patients according to our literature search. In a cohort of patients with HF, whole blood concentrations of C16:1n-7t were slightly higher (0.29% ± 0.20) than in our cohort [29]. We observed that blood levels of C16:1n-7t were inversely associated with metabolic indices indicative of cardiometabolic risk such as triglycerides-to-HDL-C ratio, triglycerides, body mass index, waist circumference and biochemical markers of non-alcoholic fatty liver disease (NAFLD) at baseline. Furthermore, higher blood levels of C16:1n-7t were predictive of these markers after 12mFU. This observation, which overall indicates a more favorable metabolic phenotype with higher circulating levels of C16:1n-7t broadly aligns with the existing literature. For example, plasma biomarkers (i.e., certain saturated fatty acids) of full fat dairy (which is also a major source of naturally occurring TFA) have been linked to lower cardiometabolic risk factors and to lower cardiovascular disease risk [28, 30, 31]. With respect to circulating plasma levels of the ruminant TFA C16:1n-7t specifically, a neutral association with total mortality has been described [32]. Kleber et al. [16] reported that higher red blood cell (RBC) levels of TFA C16:1n-7t were associated with reduced mortality risk in patients referred for coronary angiography from the Ludwigshafen Risk and Cardiovascular Health Study (LURIC).

#### Industrially processed TFA

IP-TFA (trans oleic acid (C18:1n9t) and the three trans linoleic (C18:2n6) isomers) are formed during the industrial process of partial hydrogenation of vegetable oils or deep-frying [8]. In the Aldo-DHF cohort, mean whole blood percentages of the IP-TFA C18:1n9t, C18:2n6tt, C18:2n6ct and C18:2n6tc expressed as a percentage of a total of 26 identified FAs in whole blood were 0.51%, 0.01%, 0.04% and 0.12%, respectively. According to our literature search, there are no comparable data available on whole blood TFA concentrations in HFpEF patients, but in a cohort of patients with HF, whole blood concentrations were similar as in our population. [29].

### Lipid phenotype

Higher blood levels of trans oleic acid C18:1n9t and the trans linoleic acid isomer C18:2n6tc were associated with higher non-HDL-C and LDL-C at baseline, which are both established cardiovascular risk factors, the former being a better predictor for cardiovascular endpoints in individuals with MetS and T2D [33]. In addition, higher trans oleic acid C18:1n9t was associated with metabolic markers of a higher risk lipid and plaque phenotype such as triglycerides-to-HDL-C ratio [34–36] and triglycerides [37] at baseline.

### Glucose metabolism

The trans linoleic acid isomers C18:2n6tt and C18:2n6ct were predominantly associated with higher HbA1c at baseline. T2D is a common comorbidity with prognostic impact in patients living with HFpEF and necessitates comprehensive management [1, 38]. This may, among other reasons, provide one biologically plausible explanation for the observed benefit of the oral antidiabetic drug SLGT2i Empagliflozin in patients with HFpEF [6]. Sensitivity analyses with medication group as covariate showed significant effects of group allocation (spironolactone^+/−^) for the association between TFA and the 12mFU outcome HbA1c, possibly indicating that the study medication confounded the direct association between IP-TFA status and HbA1c at 12mFU.

### Inflammation

IP-TFA foster a systemic milieu characterized by adipose tissue, vascular and systemic inflammation [8, 11] which is an intermediate risk factor for adverse outcomes in HFpEF [1]. Mozaffarian and colleagues [19] showed that in 86 ambulatory patients with established heart failure, RBC TFAs were strongly associated with NT-proBNP and biomarkers of systemic inflammation (i.e., IL 1β, IL-1 receptor antagonist, IL-6, IL-10, TNF, TNF receptor 1 and 2, monocyte chemoattractant protein 1). Regarding markers of inflammation, in our cohort, leukocytes were available as a broad and unspecific marker of inflammatory status. While leukocytes showed no association with IP-TFA, interestingly, higher blood levels of the naturally occurring C16:1n-7t were consistently associated with lower leukocyte count at baseline and prognostic for lower leukocyte count after 12mFU. One hypothesis that may offer some biological plausibility for this observation is that blood levels of naturally occurring trans-fatty acids are a biomarker, e.g., intake of dairy fat. Dairy and its components influences diverse pathways related to cardiometabolic health (reviewed in [31]), including oxidative and inflammatory pathways, intestinal integrity and endotoxemia related to broad inflammation [31].

### Neurohumoral activation and cardiac function

As opposed to the findings of Mozaffarian et al. [19] we observed a significant positive association with neurohumoral activation for only one of the three IP-TFA trans linoleic (C18:2n6) isomers (C18:2n6tc). This association persisted after 12mFU. However, the strength of the association was very weak and such an association was not observed for all other IP-TFA. Reasons for this inconsistency may be the different methodology used (red blood cell TFA in the analysis of Mozaffarian et al. vs whole blood TFA in our analysis) and the different study populations (HF patients in the analysis of Mozaffarian et al. vs HFpEF patients in our analysis). No association between blood TFAs and echocardiographic markers of left ventricular diastolic dysfunction were observed in the Aldo-DHF cohort.

### Functional capacity

Higher blood levels of the trans linoleic acid isomers C18:2n6tt and C18:2n6ct were very consistently associated with lower submaximal aerobic capacity at baseline and at 12mFU. Functional capacity is an important prognostic marker in HF/HFpEF [3]. Therefore, its inverse association with trans linoleic acid isomers C18:2n6tt and C18:2n6ct may be a clinically relevant finding that (a) warrants future studies to substantiate these findings and (b) may justify discouraging HFpEF patients from consuming foods that contain trans fats. According to our literature search, no data are available on the association of IP-TFA and functional capacity in HFpEF patients.

### Translational perspective

Partially hydrogenated vegetable oils are attractive ingredients for the food industry due to their long shelf life, their stability during deep-frying and their semisolid texture, which is used to enhance the palatability of baked goods and sweets [8]. Due to uncontroversial evidence on their adverse health effects, in the USA, regulatory agencies pressured dietary oil producers to remove IP-TFA from their products. In the USA, this has resulted in declining IP-TFA levels in red blood cells since 1999 [39] and between 2009 and 2016 has collinearly led to the desired effect of declining rates of fatal ischemic heart disease (*r* = 0.9552, *p* < 0.0001) [40]. Similarly, in Europe, from 2008 to 2015, levels of IP-TFA and ruminant-derived C16:1n-7t, a marker for dairy and meat intake, have been decreasing with few individuals having levels of IP-TFA above a safe range, while many had low levels of C16:1n-7t [41]. In line, Kleber et al. [16] suggested that TFA levels up to 1.04% of total fatty acids of erythrocytes membranes might be regarded as safe and are not associated with adverse health outcomes. This overall suggests a general decline in TFA exposure due to policy changes [18]. However, it is worth noting that, even within the low levels of IP-TFA blood levels observed in the Aldo-DHF cohort, there was a consistent positive association with high-risk metabolic traits in HFpEF patients (whole blood TFA acids in our cohort, as compared to erythrocyte TFA acid levels in the analysis by Kleber et al. [16], were lower by a potency of 10 but similar to whole blood TFA concentrations in another German cohort with HF [29]). Therefore, despite the limitations of observational data in addressing causation questions, our findings support the notion that further efforts to removing IP-TFA from the food supply and/or stronger public health efforts to discourage consumption of food sources substantially contributing to trans fat consumption (i.e., fried foods, margarine, processed meats, bakery products and biscuits) [42] may ameliorate risk factor control in patients living with HFpEF, a condition where prognosis is largely determined by managing comorbidities [1]. Our findings reflect the differential association of individual TFA sources (industrial vs. natural ruminant) on HFpEF phenotype. In line with the literature, this may speak to encouraging increased consumption of whole foods such as full fat dairy that contain naturally occurring TFA or potentially supplementing the naturally occurring TFA C16:1n-7t, for improving cardiometabolic health also in HFpEF patients [28, 43]. Furthermore, these findings support the notion that trans-fatty acids, like saturated fatty acids [44] and omega-3 fatty acids [21], are a heterogeneous group with respect to health interactions. This overall speaks to considering individual fatty acids instead of referring to predefined groups of fatty acids based on their physicochemical properties.

### Limitations and strengths

A strength of this analysis is the large, well phenotyped cohort comprising 404 patients with HFpEF. Second, 53% of the patients included in Aldo-DHF were female, resulting in a representative gender distribution for the condition HFpEF, which is slightly more common in females [45]. Third, blood fatty acid levels are an objective biomarker of TFA status unbiased by recall-based dietary intake assessment methods (e.g., food frequency questionnaires) used in nutritional epidemiology which may not accurately reflect an individual’s consumption of nutrients due to limitations such as measurement error, recall bias, selective reporting and incompleteness of food composition databases [22].

Our analysis is not without limitations. First, our analysis does not offer information on the prognostic impact of blood TFA levels in patients with HFpEF. We analyzed associations of TFA blood levels and surrogate markers for HFpEF prognosis. Future research is warranted to determine the prognostic relevance of our findings. Second, this is an observational study that cannot address causation questions. Recognizing this limitation, our findings may support our hypothesis that efforts to remove IP-TFA from the food supply in Europe may be beneficial for patients with HFpEF. Third, little is known about the efflux of TFA blood levels, i.e., the relative contribution and determinants of elimination of these fatty acids on actual blood levels. Fourth, since 1 April 2021, the European Parliament has forbidden the marketing of foods that contain trans fat from industrial origin (excluding naturally occurring in fat of animal origin) of more than 2 g per 100 g of fat (2%) (https://ec.europa.eu/food/safety/labelling-and-nutrition/trans-fat-food_en). This may have led to declining levels of blood IP-TFA since data acquisition for the Aldo-DHF study (March 2007–April 2012). Current blood IP-TFA in HFpEF patients are not known. Finally, in the Aldo-DHF study, no data were obtained on dietary intake, and therefore, we cannot provide data on diet or related variables such as macronutrient distribution. In this regard, it is, however, worth noting that blood levels of fatty acids are determined not only by intake, but also by bioavailability, distribution volume, catabolism and other factors (e.g., de novo lipogenesis of saturated fatty acids from carbohydrates in the context of insulin resistance/non-alcoholic fatty liver disease). Therefore, circulating levels of fatty acids represent the status of an individual in these fatty acids—and as has been repeatedly demonstrated, the status of an individual of any given fatty acid correlates more closely with clinical events or other biological parameters than intake. Therefore, from the perspective of scientific rigor, reporting levels of fatty acids as an objective scientific metric but not dietary intake (as assessed by subjective memory-based methods) may alternatively be seen as a strength of our study.

## Conclusions

In HFpEF patients, higher blood levels of industrially processed trans-fatty acid subtypes including trans oleic (C18:1n9t), and the three trans linoleic (C18:2n6) isomers were associated with a higher risk phenotype, whereas the naturally occurring TFA trans-palmitoleic acid (C16:1n-7t) occurring in full fat dairy and meat was associated with a lower risk phenotype in patients with HFpEF. Albeit the limitations of observational data in addressing causation questions, our findings suggest that further efforts to remove industrially processed trans-fatty acids from the food supply may be helpful for risk factor management in patients living with HFpEF. Furthermore, blood TFAs, in particular C16:1n-7t, warrant further investigation as prognostic markers in HFpEF.

### Supplementary Information

Below is the link to the electronic supplementary material.

Supplementary file1 (DOCX 32 KB)

## Data Availability

Due to the GDPR regulations and local data protection laws we cannot provide patient data.
