# Supplementary material for: Trans-fatty acid blood levels of industrial but not natural origin are associated with cardiovascular risk factors in patients with HFpEF: a secondary analysis of the Aldo-DHF trial
Source: Clin Res Cardiol. 2023 Jan 14;112(11):1541–54. doi: 10.1007/s00392-022-02143-7 (PMC10584704; doi:10.1007/s00392-022-02143-7)
Supplement: Supplementary file 1 — Supplementary file1 (DOCX 32 KB) [file 392_2022_2143_MOESM1_ESM.docx]

**Supplemental Table 1: C**orrelations adjusted for BMI, markers for truncal adiposity (waist circumference and waist-to-height ratio), HbA1c, and systolic/diastolic blood pressure between individual TFA and patient characteristics at baseline

|  |  | C16:1n-7t (trans-palmitoleic acid) | C18:1n9t | C18:2n6tt | C18:2n6ct | C18:2n6tc |
| --- | --- | --- | --- | --- | --- | --- |
| LDL-C | r^§^  p-value* | 0,056  0,282 | **0,103**  **0,048** | **0,118**  **0,024** | 0,08  0,123 | **0,135**  **0,009** |
| non-HDL-C | r^§^  p-value* | -0,014  0,786 | 0,054  0,298 | **0,126**  **0,015** | **0,104**  **0,046** | 0,094  0,072 |
| triglycerides | r^§^  p-value* | **-0,158**  **0,002** | 0,024  0,644 | 0,038  0,463 | 0,031  0,553 | 0,029  0,572 |
| triglycerides-to-HDL-C ratio | r^§^  p-value* | **-0,176**  **0,001** | 0,011  0,831 | 0,021  0,68 | 0,004  0,933 | 0,026  0,623 |
| ASAT | r^§^  p-value* | -0,022  0,666 | 0,004  0,94 | -0,007  0,893 | -0,012  0,811 | 0,063  0,229 |
| ALAT | r^§^  p-value* | **-0,124**  **0,017** | -0,087  0,095 | -0,065  0,212 | -0,045  0,386 | -0,006  0,906 |
| GGT | r^§^  p-value* | -0,098  0,061 | -0,084  0,105 | 0,075  0,147 | 0,035  0,504 | 0,004  0,936 |
| distance covered 6 MWT | r^§^  p-value* | -0,045  0,387 | -0,066  0,205 | -0,068  0,188 | -0,052  0,318 | -0,002  0,97 |
| VO2peak | r^§^  p-value* | -0,03  0,559 | -0,072  0,168 | 0,031  0,549 | 0,092  0,077 | 0,051  0,327 |
| E/e' | r^§^  p-value* | -0,063  0,226 | 0,005  0,93 | -0,032  0,537 | -0,034  0,513 | -0,056  0,278 |
| NT-proBNP | r^§^  p-value* | 0,019  0,714 | 0,068  0,193 | 0,046  0,375 | 0,025  0,637 | **0,113**  **0,03** |

Abbreviations: NT-proBNP (N-terminal pro–braintype natriuretic peptide), GGT (γ-glutamyltransferase), ASAT (aspartate aminotransaminase), ALAT (alanine aminotransaminase), E/e´ (diastolic function), VO2peak (maximum exercise capacity). Significant values are in bold. *All tests were performed 2-sided. r^§^ (Spearman’s correlation coefficient).

**Supplemental Table 2:** Correlations adjusted for BMI, markers for truncal adiposity (waist circumference and waist-to-height ratio), HbA1c, and systolic/diastolic blood pressure between individual TFA and patient characteristics at 12mFU

|  |  | C16:1n-7t (trans-palmitoleic acid) | C18:1n9t | C18:2n6tt | C18:2n6ct | C18:2n6tc |
| --- | --- | --- | --- | --- | --- | --- |
| LDL-C | r^§^  p-value* | 0,06  0,269 | **0,158**  **0,003** | 0,063  0,248 | 0,015  0,78 | 0,072  0,187 |
| non-HDL-C | r^§^  p-value* | 0,034  0,53 | 0,082  0,13 | 0,098  0,071 | 0,041  0,446 | 0,054  0,318 |
| triglycerides | r^§^  p-value* | **-0,121**  **0,025** | 0,01  0,853 | 0,042  0,437 | 0,008  0,884 | 0,004  0,941 |
| triglycerides-to-HDL-C ratio | r^§^  p-value* | **-0,151**  **0,005** | -0,013  0,804 | 0,029  0,59 | -0,031  0,572 | -0,019  0,722 |
| ASAT | r^§^  p-value* | -0,04  0,462 | 0,038  0,483 | 0,039  0,472 | 0,069  0,201 | 0,079  0,147 |
| ALAT | r^§^  p-value* | **-0,153**  **0,005** | -0,054  0,324 | 0,002  0,97 | -0,042  0,436 | -0,033  0,538 |
| GGT | r^§^  p-value* | **-0,143**  **0,008** | -0,032  0,556 | 0,031  0,564 | 0,013  0,81 | -0,051  0,35 |
| distance covered 6 MWT | r^§^  p-value* | -0,031  0,567 | 0,024  0,654 | **-0,113**  **0,036** | **-0,119**  **0,028** | 0,021  0,697 |
| VO2peak | r^§^  p-value* | -0,075  0,17 | -0,048  0,379 | -0,106  0,051 | -0,044  0,413 | 0,047  0,382 |
| E/e' | r^§^  p-value* | -0,009  0,873 | -0,027  0,614 | -0,041  0,447 | -0,09  0,098 | -0,097  0,073 |
| NT-proBNP | r^§^  p-value* | 0,082  0,133 | 0,046  0,401 | 0,058  0,289 | 0,066  0,225 | **0,113**  **0,038** |

Abbreviations: NT-proBNP (N-terminal pro–braintype natriuretic peptide), GGT (γ-glutamyltransferase), ASAT (aspartate aminotransaminase), ALAT (alanine aminotransaminase), E/e´ (diastolic function), VO2peak (maximum exercise capacity). Significant values are in bold. *All tests were performed 2-sided. r^§^ (Spearman’s correlation coefficient).
